# Energy expenditure in myelofibrosis patients treated with a JAK1/2 inhibitor

**DOI:** 10.3389/fendo.2023.1141029

**Published:** 2023-06-29

**Authors:** Douglas Tremblay, Mikaela Dougherty, John Mascarenhas, Emily Jane Gallagher

**Affiliations:** ^1^ Tisch Cancer Institute, Icahn School of Medicine at Mount Sinai, New York, NY, United States; ^2^ Division of Endocrinology, Diabetes, and Bone Diseases, Department of Medicine, Icahn School of Medicine at Mount Sinai, New York, NY, United States

**Keywords:** ruxolitinib, energy expenditure, weight gain, intolerance, leptin

## Abstract

Weight gain is a known adverse effect of ruxolitinib, a JAK1/2 inhibitor that is the mainstay of treatment for many patients with myelofibrosis. The mechanisms behind weight increase with ruxolitinib is incompletely understood, although decreased adipose tissue lipolysis and increased appetite due to blocking the effects of leptin in the hypothalamus have been proposed. In order to explore the metabolic changes in ruxolitinib-treated patients with myelofibrosis, we performed a pilot study to assess the feasibility of using a portable indirect calorimeter to quantify energy expenditure before and during ruxolitinib treatment and report the results of two patients. Waist circumference increased during ruxolitinib treatment in both patients. Energy expenditure initially increased followed by a decrease and then increase again, but to levels below baseline. These results suggest that weight gain secondary to ruxolitinib may be related to changes in whole body energy expenditure.

## Introduction

Myelofibrosis (MF) is a hematologic malignancy clinically characterized by abnormal cytokine production, splenomegaly, and constitutional symptoms that include weight loss (1). Ruxolitinib (Jakafi, Incyte) is a JAK1/2 inhibitor and was the first Food and Drug Administration approved therapy for MF, based on the results of the COMFORT-I and -II studies that demonstrated efficacy in terms of spleen reduction and symptoms improvement (2, 3). Interestingly, ruxolitinib is associated with statistically and clinically significant weight gain. In the pivotal, phase 3 COMFORT-I and COMFORT-II trials, patients treated with ruxolitinib gained an average of 3.9 kg after 24 weeks (COMFORT-I) and 4.4 kg after 48 weeks (COMFORT-II) of ruxolitinib therapy (2–4). Weight gain occurs regardless of pretreatment body mass index (BMI), suggesting weight gain is not solely attributable to resolution of MF symptoms (e.g., early satiety, abdominal discomfort) in cachectic patients (5).

We have previously shown in a retrospective analysis of 179 patients with MPNs treated with ruxolitinib that 69% of patients experienced weight gain during treatment and the average gain for those patients was 12% of pretreatment body weight, with over 50% of patients gaining > 5% of their pretreatment body weight (5). There is uncertainty as to the pathogenesis of ruxolitinib-mediated weight gain. In a murine model, ruxolitinib reduced the normal JAK2/STAT3 phosphorylation in neuronal cells in mouse hypothalamus that occurs in response to feeding or to administration of exogenous leptin, which typically signals satiety, suggesting that increased appetite may be responsible (5). However, we have also demonstrated that ruxolitinib inhibits JAK/STAT signaling in the adipose tissue, which is an important regulator of adipose tissue lipolysis (6, 7).

To gain further insight into the metabolic changes that occur during ruxolitinib therapy, we performed a pilot and feasibility study of patients with MF using a portable indirect calorimeter to determine energy expenditure before and during treatment with ruxolitinib. Here, we describe two MF patients that highlight metabolic changes with this JAK1/2 inhibitor.

## Methods

MF patients without prior JAK inhibitor exposure who were intending to start ruxolitinib as standard of care therapy were prospectively enrolled. Patients with chronic obstructive pulmonary disease, untreated endocrinopathy aside from diabetes, heart failure, or additional malignancies were excluded. Enrolled patients had resting energy expenditure measured by indirect calorimetry at screening and an additional baseline visit. Patients then had repeat measurements performed 2, 4, 8, 16, and 24 weeks after starting ruxolitinib. Weight, height, and waist circumference were recorded at each study visit, in addition to standard of care laboratory evaluation. A portable indirect calorimeter was kindly provided by PNOE (Palo Alto, California) (8). This device includes a wearable facemask, where the subject breathes through the Micro-Electro-Mechanical Systems based hot film anemometer flow sensor that operates on a breath-by-breath mode that continuously measures volume and determines expired gas concentrations simultaneously. Heart rate was measured using a heart rate monitor (Polar Electro, Lake Success, NY). Respiratory and cardiac measurements were transmitted *via* telemetry. Patients were required to fast for at least 8 hours prior to the testing, and not to exercise or use nicotine containing products for at least 4 hours prior to testing. They were seated at rest for at least 10 minutes before measurements were taken.

The Myeloproliferative Neoplasms Symptom Assessment Form Total Symptom Score (MPN-SAF-TSS) was calculated at each visit (9). The MPN-SAF-TSS includes ten symptoms: fatigue; early satiety; abdominal discomfort; inactivity; impaired concentration; night sweats; pruritis; bone pain; fever; unintentional weight loss over preceding 6 months. Each symptom is rated on a scale of 0-10, with 0 being absence of symptoms, and 10 being the worst imaginable symptoms. The MPN-SAF-TSS has a range from 0-100, with 100 being the most severe symptom severity.

All subjects signed a written informed consent prior to their participation in the study. The study was approved by the Program for the Protection of Human Subjects Institutional Review Board at the Icahn School of Medicine at Mount Sinai. All procedures were in accordance with the Declaration of Helsinki.

## Results

### Baseline characteristics

We report observations from two patients enrolled in this study. Additional patient enrollment was ceased because of the COVID-19 pandemic. Baseline metabolic characteristics are shown in **Table 1**. While Patient 1 was normal weight with a BMI of 20.5 kg/m^2^, Patient 2 had Class III obesity (BMI 40.6 kg/m^2^) and a waist circumference of 122 cm. Both patients were newly diagnosed and treatment naive. Patient 1 had baseline leukocytosis with a white blood cell (WBC) count of 94.1 x 10^9^/L, hemoglobin of 8.9 g/dL and platelet count of 172 x 10^9^/L while Patient 2 had a WBC of 9.8 g/dL, hemoglobin of 7.3 g/dL and a platelet count of 389 x 10^9^/L. The Dynamic International Prognosis Scoring System (DIPSS) risk category was high and intermediate-2 for Patient 1 and Patient 2, respectively. Both patients were symptomatic with an MPN-SAF-TSS) of 31 and 49, respectively. Patient 1 had a baseline palpable spleen length of 15cm below the left costal margin while Patient 2 did not have palpable splenomegaly. Both patients were initiated ruxolitinib at 5mg twice daily, a dose selected considering baseline disease-related anemia.

**Table 1:** Baseline patient characteristics.

	Patient 1	Patient 2
**Age/sex**	67 male	40 female
**Race/Ethnicity**	Black	Hispanic
**Comorbidities**	Chronic photosensitive dermatitis	Coronary artery disease, hypothyroidism (controlled)
**Diagnosis**	Primary myelofibrosis	Post-essential thrombocythemia myelofibrosis
**DIPSS risk category**	High risk	Intermediate-2
**Driver mutational status**	Triple negative	Triple negative
**Palpable spleen length below left costal margin (cm)**	15	0
**Weight (kg)**	75.4	110.6
**BMI (kg/m^2^)**	20.5	40.6
**Waist circumference (cm)**	99.5	122
**Albumin (g/dL)**	3.3	3.5
**LDL (mg/dL)**	50	70
**HDL (mg/dL)**	11	22
**Triglycerides (mg/dL)**	85	171
**LDH (units/dL)**	810	473

### Changes in metabolic parameters during treatment

The body weight of Patient 1 increased from 75.6kg to 83.9kg at 24 weeks, a gain of 8.3kg. Patient 2 experienced mild weight loss, with a change from110.8kg to 109.3kg at 24 weeks, a loss of 1.5kg. However, waist circumference increased in both patients: Patient 1 increased from 99.5cm at baseline to 104cm at 24 weeks, and Patient 2 increased from 122cm to 128cm at 24 weeks.

Indirect calorimetry readings displayed interesting dynamics in both patients. The resting energy expenditure for Patient 1 initially increased from a baseline of 3158 kcal/day to 3355 kcal/day at week 2 and then dropped to 2303 kcal/day at week 4 before rebounding to 2686 kcal/day at week 8 and then plateaued. Patient 2 had a baseline resting energy expenditure of 1870 kcal/day which increased to 2530 kcal/day on week 4 before decreasing to 1420 kcal/day on week 8 before ending at 1983 kcal/day at week 24.

**Figure 1:**
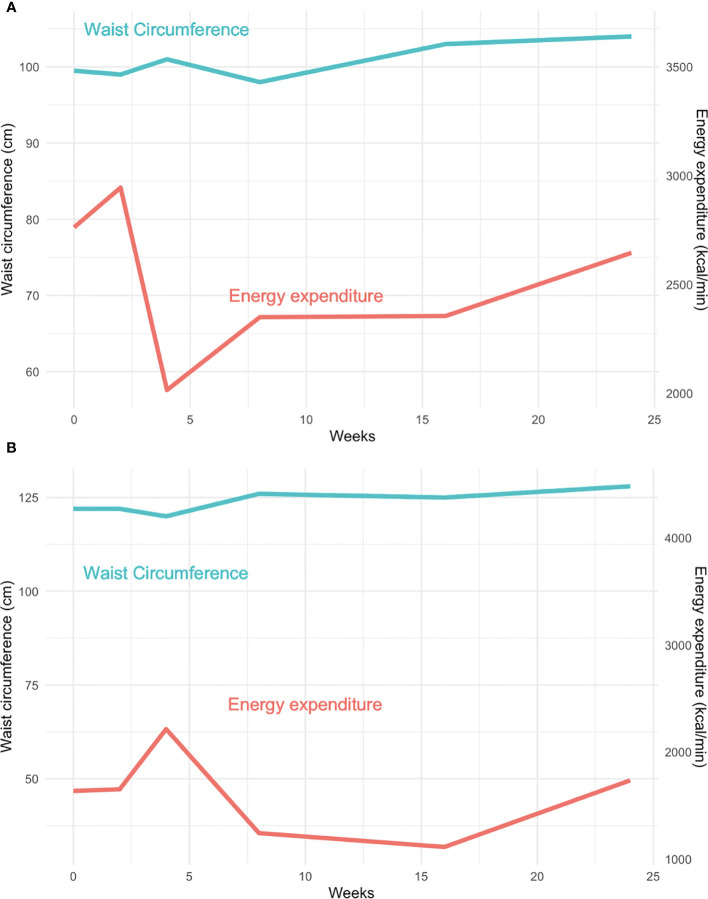
Changes in energy expenditure and waist circumferences over time. For patient 1 **(A)** and 2 **(B)**, energy expenditure initially increased after initiation of ruxolitinib then decreased which was followed by an increase in waist circumference.

**Figures 1A, B** show the energy expenditure over time plotted with changes in waist circumference. In both cases, there was an initial increase in resting energy expenditure followed by a decrease followed by an increase before returning to levels below baseline.

Changes in hemoglobin levels over time somewhat mirrored energy expenditure with initial decrease followed by an increase (**Supplemental Figure 1**).

Consistent with prior reports, albumin increased in both patients, with Patient 1 increasing from 3.3 g/dL to 3.9 g/dL at week 24 and increasing from 3.6 g/dL to 3.7 g/dL at week 24. Triglyceride levels also increased from a baseline of 85 mg/dL to 143 mg/dL and 171 mg/dL to 259 mg/dL for Patient 1 and 2, respectively. Patient reported outcome measure of early satiety during the treatment course steadily decreased from 8 out of 10 at baseline to 5 at week 24 in Patient 1, and from 8 to 7 in Patient 2.

## Discussion

Our results suggest that weight gain due to ruxolitinib may not only be mediated by changes in appetite due to impaired JAK/STAT signaling in the hypothalamus, but may also related to changes in whole body energy expenditure.

In this pilot study, we used a portable indirect calorimeter, which allowed us to bring the device to the patients, facilitating point of care testing in the oncology rather than requiring separate visits to a metabolic testing location. Having the ability to perform indirect calorimetry in the oncology center could allow us to perform larger future studies to develop a greater understanding of the links between systemic metabolism and cancer.

Leptin receptors are found not only in the hypothalamus, but are expressed in other parts of the brain, and also in other tissues including the liver, spleen, kidneys, and adipose tissue. Leptin signaling in the brain and in adipose tissue has been found to increase energy expenditure in pre-clinical models (10). Interestingly, in pre-clinical models with intact leptin signaling in the hypothalamus, but adipose tissue leptin receptor deficiency, mice gained weight despite normal food intake, and developed insulin resistance and hypertriglyceridemia (11). Growth hormone binding to the growth hormone receptor also activates the JAK/STAT pathway. Interestingly, both chronic growth hormone excess and deficiency have been associated with increased energy expenditure (12). People with growth hormone receptor deficiency have higher percent body fat; however, in contrast to the leptin deficient models, do not develop insulin resistance or hypertriglyceridemia. Therefore, the effects of ruxolitinib on energy expenditure and triglycerides may be mediated through the leptin receptor. Interestingly, none of the previous studies have found biologically significant hyperglycemia with ruxolitinib despite the weight gain. This lack of hyperglycemia could be related to inhibition of the antagonistic effects of growth hormone on insulin signaling.

Ruxolitinib treatment has also been shown to increase muscle mass in MF patients (13). It is possible that this increase in muscle mass may be responsible for return of energy expenditure to near baseline as skeletal muscle is a significant driver of resting energy expenditure (14). Unfortunately, abdominal imaging was not obtained in our study and thus we are unable to explore this possibility.

Our study has several limitations that limit generalizability, including the fact that both patients were negative for MPN driver mutations, a rare subset of MF patients. In addition, the limited sample size and observational nature of our study did not allow us to evaluate the impact of ruxolitinib dosing on metabolic parameters. Overall, this pilot study shows the potential value of portable indirect calorimeters for measuring energy expenditure in individuals with malignancies, and suggests that the changes in energy expenditure may contribute in part to the weight gain associated with ruxolitinib.

## Author contributions

DT, JM and EG designed the study, enrolled the patients, performed the indirect calorimetry and wrote the manuscript. MD performed regulatory functions and assisted with enrollment and design of the study. All authors contributed to the article and approved the submitted version.
