# Supplementary figures and images for: Energy expenditure in myelofibrosis patients treated with a JAK1/2 inhibitor
Source: Front Endocrinol (Lausanne). 2023 Jun 29;14:1141029. doi: 10.3389/fendo.2023.1141029 (PMC10339379; doi:10.3389/fendo.2023.1141029)

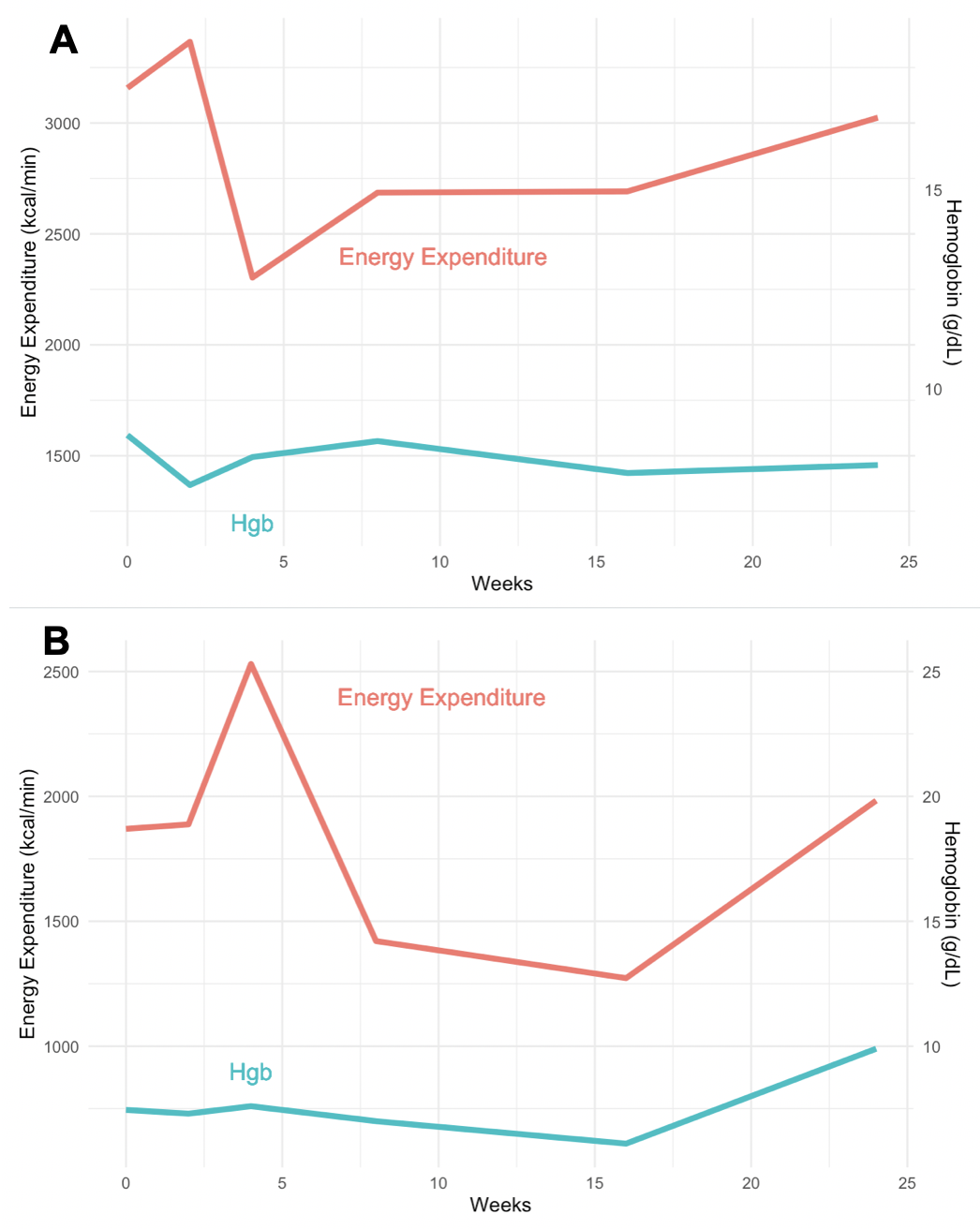

Supplement: Supplementary file 1 [file Image_1.tiff]
